# Distinct VE-cadherin serine and tyrosine phosphorylation sites and their role for inflammation-induced vascular permeability in vivo

**DOI:** 10.1007/s00018-025-05753-2

**Published:** 2025-06-05

**Authors:** Leonie Holtermann, Ronmy Rivera-Galdos, Astrid F. Nottebaum, Florian Wessel, Ute Ipe, Dietmar Vestweber

**Affiliations:** Present Address: Department of Vascular Cell Biology, Max Planck Institute for Molecular Biomedicine, Röntgenstrasse 20, 48149 Münster, Germany

**Keywords:** Endothelium, Junction regulation, VE-cadherin mutants, Protein modification, Signaling

## Abstract

VE-cadherin is a major component of endothelial adherens junctions and pivotal to the regulation of vascular barrier integrity. Whereas two phosphorylation sites of VE-cadherin (Y685 and Y731) are known to be relevant for the regulation of endothelial junctions in vivo, several others were suggested to be relevant based on in vitro studies. Here, we analyze for two of these, serine 665 (S665) and tyrosine 658 (Y658), whether they are relevant for the induction of vascular permeability in vivo. To this end, we generated and characterized two point-mutated VE-cadherin knock-in mouse lines where either S665 was replaced by valine (S665V) or Y658 by phenylalanine (Y658F). We found that the induction of vascular permeability by histamine or VEGF in the skin was clearly reduced in S665V mice, whereas Y658F mice showed a normal increase of permeability. In line with this, we found that histamine-induced endocytosis was impaired for the VE-cadherin-S665V mutant, but not for the Y658F mutant. Comparing the regulation of VE-cadherin phosphorylation at S665, Y658 and Y685, we found that only phosphorylation of S665 and Y685 were strongly induced by inflammatory mediators, while phosphorylation of Y658 increased weakly. Interestingly, phosphorylation of S665 and Y685 occurred with different kinetics, but independent of each other. Collectively, our results demonstrate that Y658 is irrelevant for vascular leak formation in the context of several tested inflammatory mediators and establish S665 of VE-cadherin as an important phosphorylation site regulating the induction of endothelial permeability in vivo*.*

**Supplementary Information:**

The online version contains supplementary material available at 10.1007/s00018-025-05753-2.

## Introduction

Vascular integrity is maintained by the endothelium, which provides a barrier between blood and interstitium. In many pathological scenarios, inflammatory mediators compromise the endothelial barrier, leading to increased vascular permeability and hence, plasma leakage. This requires interference with junction integrity between endothelial cells, a process that targets the cell adhesion molecule VE-cadherin.

VE-cadherin is a major component of endothelial adherens junctions and pivotal to the regulation of vascular barrier integrity. Antibody-mediated blockade of the adhesive function of VE-cadherin increases endothelial permeability in vitro and in vivo [1–3]. VE-cadherin is stabilized at endothelial junctions via linkage to the actin cytoskeleton, which is mediated by catenins [4, 5] and additional adaptor proteins such as vinculin and EPLIN [6, 7]. Increasing the adhesiveness of VE-cadherin in vivo by enhancing its interaction with α-catenin or the phosphatase VE-PTP suppresses vascular leak formation and leukocyte extravasation in appropriate knock-in mice [8, 9].

The integrity of endothelial junctions was furthermore found to be modulated by phosphorylation of catenins [10–12] and VE-cadherin itself [13–15]. Several tyrosine residues in the cytoplasmic domain of VE-cadherin were suggested to regulate either endothelial permeability or the extravasation of leukocytes. Y685 was identified as a direct target of Src kinase in VEGF-stimulated endothelial cells in vitro [16]. In vivo, Y685 was found to be phosphorylated in veins but not arteries in a shear force and Src-dependent manner [17]. To directly test whether tyrosine phosphorylation of VE-cadherin was indeed required in vivo for the regulation of endothelial junctions, we generated point-mutated knock-in mice where either Y685 or Y731 of VE-cadherin were replaced by phenylalanine. With these mice we could show that Y685 phosphorylation was crucial for the induction of vascular permeability in response to VEGF or histamine but dispensable for the process of leukocyte extravasation, whereas Y731 proved to be relevant only for the diapedesis process [18]. Mechanistically, bradykinin was shown to cause the endocytosis of phosphorylated VE-cadherin in vitro, in a Y685-dependent manner [17]. This correlated with increased ubiquitination of VE-cadherin. Recently, we demonstrated that histamine-induced ubiquitination of VE-cadherin occurs at two membrane-proximal lysine residues, K626 and K633. These modifications occurred downstream of the phosphorylation of Y685 and were indeed required for histamine-induced internalization of VE-cadherin, and, consequently, for the induction of vascular permeability in vivo [19].

Reports on the role of Y658 of VE-cadherin for vascular permeability regulation are controversial. A study by Orsenigo et al. showed that, similar to Y685, tyrosine 658 was only phosphorylated in veins and not in arteries and that the Y658F mutation inhibited bradykinin-induced VE-cadherin endocytosis and paracellular permeability across cultured endothelial cells [17]. In contrast, another study claimed that mutating Y658 of VE-cadherin did not affect endothelial permeability induction in vitro [20].

Phosphorylation sites in junction-associated proteins that regulate adherens junction dynamics are not limited to tyrosine residues [21]. One such site is serine 665 in the cytoplasmic domain of VE-cadherin. It was reported that VEGF-induced activation of Src does not only lead to direct phosphorylation of Y685 of VE-cadherin [16], but also stimulates, indirectly, the phosphorylation of S665 via a complex signaling cascade comprising the RhoGEF Vav2, Rac1 and the serine/threonine kinase PAK1 [22]. Based on a non-phosphorylatable VE-cadherin mutant carrying a valine at the position of S665 (S665V), the study demonstrated that phosphorylation of S665 was required for VEGF-induced VE-cadherin internalization. Mechanistically, S665 phosphorylation led to the recruitment of the scaffolding protein β-arrestin2, which initiated VE-cadherin endocytosis into clathrin-coated endosomes.

In the present study, we sought to find out whether Y658 and S665 of VE-cadherin are indeed relevant for the regulation of vascular permeability by inflammatory mediators in vivo. To this end, we generated VE-cadherin-Y658F and VE-cadherin-S665V point-mutated knock-in mouse lines. We found that, similar to our former results for Y685 [18], S665 is required for the induction of vascular permeability by histamine and VEGF in vivo, whereas Y658 is irrelevant for this process. In agreement with this, histamine-induced endocytosis of VE-cadherin was blocked by the S665V mutation, but not by the Y658F mutation. Comparing the regulation of VE-cadherin phosphorylation at S665, Y658 and Y685 directly, we found that only phosphorylation of S665 and Y685 were strongly induced by inflammatory mediators, while phosphorylation of Y658 increased only moderately. Interestingly, phosphorylation of S665 and Y685 occurred with different kinetics, but independent of each other. Thus, S665, but not Y658, is relevant in vivo for the regulation of vascular permeability in inflammation and acts together with, but independent of Y685.

## Material and methods

### Mice

The two VE-cadherin-Y658F and VE-cadherin-S665V point-mutated knock-in mouse lines were generated by recombinase-mediated cassette exchange (RMCE) essentially as described for other VE-cadherin mutants [8, 9, 18], such that endogenous VE-cadherin was replaced by the mutant constructs. The respective mutations were inserted into the cassette exchange vector U5-3, containing the VE-cadherin cDNA, a polyA transcriptional stop cassette and a hygromycin cassette flanked by FRT sites using the QuikChange Site-Directed Mutagenesis Kit (Agilent Technologies). The insertion cassette was flanked by the two incompatible LoxP and Lox2272 sites and RMCE was achieved by Cre-mediated homologous recombination of mouse embryonic stem (ES) cells in which exon 2 of VE-cadherin was flanked with the same Lox sites. Positive ES cell clones were identified by PCR and Southern Blot analysis and injected into blastocysts of C57BL/6 mice. Chimeras were backcrossed at least eight times with C57BL/6 mice and intercrossed to generate homozygous mutant offspring. Control mice were generated by inserting a VE-cadherin cDNA into the endogenous VE-cadherin locus in the same way and were characterized previously by our group [8]. Genotyping was performed as described before [8, 9]. All mice were bred under pathogen-free conditions in the animal facility of the Max Planck Institute for Molecular Biomedicine and were given food and water ad libitum. All animal experiments were carried out as approved by the Landesamt für Natur, Umwelt und Verbraucherschutz Nordrhein-Westfalen, Germany (81–02.04.2020.A187; 84–02.04.2014.A118).

### In vivo skin permeability assay

Vascular permeability in the skin was analyzed by a modified Miles Assay as previously described [23]. Each assay was conducted with four to seven 8- to 12-week-old female mice per group. Their dorsal skin was shaved and 100 µl of a 1% solution of Evans blue dye (Sigma-Aldrich) in PBS was injected into the tail vein. 15 min later, 50 µl PBS or 50 µl PBS containing either 100 ng VEGF or 225 ng histamine was injected intradermally into the dorsal skin. 30 min later, mice were sacrificed, skin areas were excised, and the dye was extracted with formamide (Sigma-Aldrich) for 5 days. The amount of extravasated dye was determined at 620 nm using a spectrophotometer (UV-1900i, Shimadzu).

### Whole mount immunofluorescence staining

To analyze VE-cadherin expression and junctional localization in mouse venules, whole mount staining of cremaster muscle tissue was performed. Homozygous VE-cadherin-WT, S665V or Y658F mice were sacrificed by CO_2_ asphyxiation and perfused via the left heart ventricle with 1% paraformaldehyde (PFA) in PBS. The cremaster was dissected and prefixed in situ with 4% PFA for 8 min, then removed and fixed for 1 h at RT with 1% PFA. After permeabilization and blocking in 0.3% Triton X-100, 5% donkey serum and 0.2% BSA in PBS for 2 h at RT, cremaster muscles were incubated with antibodies against PECAM-1 and VE-cadherin overnight at RT followed by Alexa Fluor 488 and Alexa Fluor 568-conjugated secondary antibodies overnight at RT, respectively. Z-stack images were acquired with a Zeiss LSM 880 confocal microscope and are depicted as maximum intensity projections.

### Antibodies

For generation of monoclonal antibody (mAb) L36.1 against phosphorylated Y658 of VE-cadherin, Lewis rats were immunized with a synthetic peptide corresponding to the respective sequence of VE-cadherin (GGEMDTTSpYDVSVLNSVR). Peptides were conjugated to a carrier protein and immunizations, mAb screening and production was performed as described elsewhere [24]. Rabbit antisera to phosphorylated S665 of VE-cadherin were raised against the peptide DVSVLNpSVRRGG conjugated to a carrier protein according to a previously described method [25]. Polyclonal antibodies (pAb) VD53 were purified from antisera by affinity chromatography. First, antisera were depleted of non-specific immunoglobulin. To this end, the unphosphorylated peptide surrounding VE-cadherin-S665 (DVSVLNSVRRGG) was immobilized on Sulfo Link Coupling Resin (Thermo Scientific) and used as affinity matrix. Depletion was repeated until the flow-through was devoid of protein (A_280_ < 0.05). Specific polyclonal antibodies were then obtained from depleted antisera by affinity purification on the corresponding phosphorylated peptide (DVSVLNpSVRRGG).

The following previously generated antibodies were used: Polyclonal rabbit antibody C5 against murine VE-cadherin [3] and monoclonal rat antibodies 1G5.1 and 5D2.6 against murine PECAM-1 [23] have been described previously.

The following monoclonal antibodies were purchased: against human VE-cadherin: Alexa Fluor 647-coupled 55-7H1 (561567, BD Pharmingen), D-87F2 XP (#2500, Cell Signaling) and F-8 (sc-9989, Santa Cruz), against α-tubulin (T6074, Sigma-Aldrich), against α-catenin (610194, BD Pharmingen), against β-catenin (610154, BD Pharmingen), against γ-catenin (610254, BD Pharmingen), against phospho-p44/42 MAPK (#9106, Cell Signaling), against p44/42 MAPK (#9102, Cell Signaling), against p120-catenin (2 F7H8, 66208–1-lg, Proteintech).

The following polyclonal antibodies were purchased: against murine VE-cadherin: AF1002 (R&D Systems) and C-19 (sc-6458, Santa Cruz), against GFP (ab6673, abcam).

Alexa Fluor 488 and 568-coupled secondary antibodies were purchased from Invitrogen. Horseradish peroxidase-conjugated secondary antibodies were purchased from Jackson ImmunoResearch. IRDye 680RD and IRDye 800CW-coupled secondary antibodies were purchased form LI-COR.

### Cell culture and reagents

Human umbilical vein endothelial cells (HUVEC) were isolated from umbilical cords (Ethics Committee of Münster University Clinic Approval 2009–537-f-S) by treatment with 1 unit/ml Dispase II (Roche) for 10 min at 37 °C in M199 medium containing 1% penicillin/streptomycin, 20% fetal bovine serum (FBS), 100 μg/ml heparin, and 3.1 μg/ml fungizone. HUVEC were cultured in EBM-2 medium supplemented with EGM-2 MV SingleQuots (Lonza) at 37 °C and 5% CO_2_ and used for experiments between passages 3 and 4. Before experiments, HUVEC were starved as indicated in EBM-2 medium containing 1% BSA overnight and treated with 100 µM histamine (Sigma-Aldrich), 1 U/ml thrombin (Calbiochem), or 50–100 ng/ml human recombinant VEGF_165_ (R&D Systems).

### RNA interference

Expression of endogenous VE-cadherin in HUVEC was silenced by transfection with CDH5 siRNA (5′-GGUUUUUGCAUAAUAAGCTT-3′, ambion). As a negative control, AllStars Negative Control siRNA (Qiagen) was used, which does not share sequence homology with any known mammalian gene. HUVEC were transfected at 60–80% confluency with 40 nM siRNA using Lipofectamine RNAiMAX (Invitrogen) or INTERFERin (Polyplus) transfection reagent according to the manufacturer’s instructions.

### Adenoviral transduction of HUVEC

The following point-mutated adenoviral constructs of human VE-cadherin were created: Y658F, S665V, and Y685F. VE-cadherin-EGFP cDNA, with the linker GCG CGA CCG GTC GCC ACC connecting the C-terminal tyrosine 784 of VE-cadherin and the starting methionine of EGFP, in the pENTR 2B plasmid, was used to introduce the abovementioned mutations via QuikChange Lightning Site-Directed Mutagenesis (Agilent Technologies). The VE-cadherin point mutant constructs were integrated into pAd/CMV/V5-DEST vectors (Gateway Technology, Invitrogen) via an LR recombination reaction. Adenovirus was produced in 293A cells (Invitrogen, R70507). HUVEC were transduced with adenovirus as described previously [18] and used in experiments 48 h later.

### Endocytosis assay

HUVEC were silenced for endogenous VE-cadherin expression by siRNA transfection and seeded on 8-well chamber slides (ibidi) coated with 100 µg/mL fibronectin (Sigma-Aldrich).

2 h after seeding, adenovirus was added for the expression of VE-cadherin-WT-EGFP and S665V or Y658F mutant constructs. 48 h later, HUVEC were serum-starved in EBM-2 medium without supplements for 3 h. Subsequently, an Alexa Fluor 647-conjugated anti-VE-cadherin antibody (55-7H1), which binds to the N-terminal extracellular domain of VE-cadherin and is known not to block cell adhesion, was added and cells were either stimulated with 100 μM histamine or vehicle for 60 min at 37 °C. Thereafter, cells were immediately fixed with 4% PFA for 10 min at RT, washed once with PBS-MC, and covered with Fluorescence mounting medium (Dako). Per assay and per condition, 20 Z-stack images were acquired with a Zeiss LSM 880 confocal microscope (20 × objective) using Airyscan mode. The number of intracellular vesicles double positive for EGFP and Alexa Fluor 647 was quantified manually using Fiji Software [26] and normalized to the number of cells analyzed.

### Immunoprecipitation and immunoblot analysis

HUVEC were lysed in lysis buffer containing 10 mM Na_2_HPO_4_/NaH_2_PO_4_, pH 6.5, 1% NP-40 substitute, 150 mM NaCl, 2 mM EDTA, and 8.3× Complete EDTA-free Protease Inhibitor (Roche) for 30 min at 4 °C. For analysis of protein tyrosine and serine phosphorylation, 1 mM Na_3_VO_4_ or 1 mM NaF, respectively, were added to the lysis buffer. Cell debris was pelleted by centrifugation at 21,000 *g*, 4 °C for 30 min after which aliquots for immunoblot analysis were taken. The residual lysate was used for immunoprecipitation with Protein G Sepharose 4 Fast Flow (GE Healthcare) coated with the respective immunoprecipitation antibody for 2 h at 4 °C.

Murine lungs were homogenized in lysis buffer using a Precellys® Evolution homogenizer with CKMix 2 ml tubes (Bertin Technologies), lysed for 3 h at 4 °C and centrifuged at 21,000 *g*, 4 °C for 30 min. Aliquots were taken for immunoblot analysis. The remaining lysate was first pre-cleared with Protein A Sepharose (Cytiva) coated with an isotype control antibody for 1 h at 4 °C after which VE-cadherin was precipitated with VE-cadherin C5 antibody-coated Protein A Sepharose at 4 °C overnight. Immunoprecipitates were washed five times with lysis buffer. Cell or lung lysates and immunoprecipitates were mixed with 3× sample buffer (200 mM Tris–HCl pH 6.8, 0% glycerol, 6% SDS, 0.1% bromophenol blue, 150 mM dithiothreitol) or 1.5× sample buffer, respectively, and boiled at 95 °C for 5 min. Proteins were separated on 6–8% SDS–polyacrylamide gels and then transferred to 0.2 µm or 0.45 µm nitrocellulose membranes (Cytiva) by wet blotting. Chemiluminescence detection was performed with a Curix 60 film developer (Agfa) or an Odyssey Fc imaging system (LI-COR Biosciences). For fluorescence detection, an Odyssey CLx imaging systems (LI-COR Biosciences) was used.

### Statistical analysis

Total sample numbers were determined on the basis of previous studies with transgenic mouse models. Immunoblot signals were quantified using Image Studio (LI-COR Biosciences). Statistical analysis was performed using GraphPad Prism 9 software (GraphPad Software Inc.). Data are shown as mean ± standard error of the mean (SEM). Statistical significance was analyzed using an unpaired two-tailed t-test, one-way analysis of variance (ANOVA) or two-way ANOVA. Outliers were identified using the ROUT method with a maximum false discovery rate of 1%. Threshold significance levels were defined as *p* < 0.05 (*), *p* < 0.01 (**), *p* < 0.001 (***), and *p* < 0.0001 (****).

## Results

### Generation of Y658F and S665V point-mutated VE-cadherin knock-in mice

**Fig. 1:**
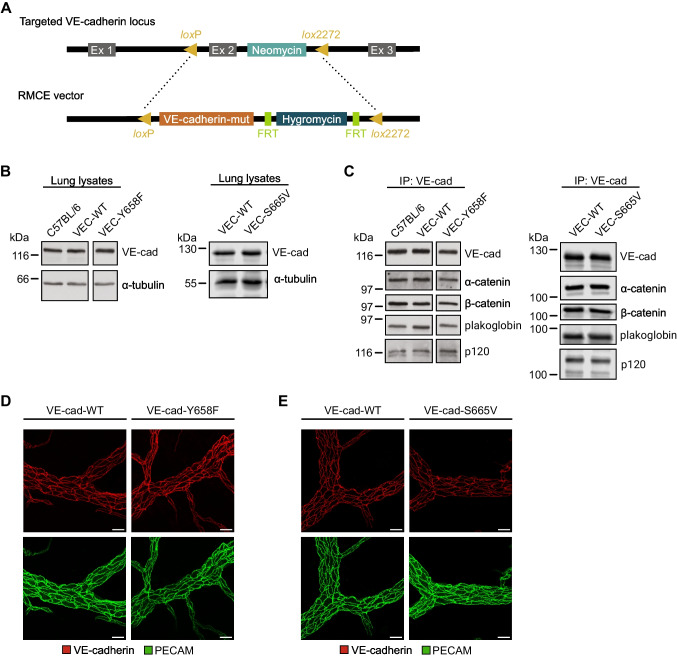
Characterization of knock-in mice expressing VEC-Y658F or VEC-S665V. **A** Replacement of the ATG-containing exon 2 (Ex 2) in the VE-cadherin locus by a replacement cassette containing cDNA encoding for Y658F or S665V mutant VE-cadherin via recombinase-mediated cassette exchange (RMCE) with two incompatible *lox* sites (*lox*P and *lox*2272). Before, exon 2 was modified using homologous recombination to flank it with the same incompatible *lox* sites. **B** Immunoblot analysis of VE-cadherin expression in lung lysates from wild-type C57BL/6 mice and homozygous VEC-WT, VEC-Y658F and VEC-S665V mice; α-tubulin serves as a loading control. **C** Immunoprecipitation of VE-cadherin from lung lysates from mice as in B, followed by immunoblotting for VE-cadherin, α-catenin, β-catenin, plakoglobin, and p120-catenin. Lanes depicting samples from C76BL/6, VEC-WT and VEC-Y658F in (B, C) are all from the same membrane. **D** + **E** Whole-mount staining of cremaster muscle from mice as in (B). Vessels were stained for PECAM-1 (red) and VE-cadherin (green). Maximum intensity projections of Z-stacks are shown. Scale bars are 25 µm. Molecular weight markers are indicated in kDa (B, C). Data are representative of at least *n = *3 independent experiments

To decipher the contribution of VE-cadherin phosphorylation at Y658 and S665 to inflammation-induced vascular permeability in vivo, we generated two different knock-in mouse lines in which endogenous VE-cadherin was replaced either by a Y658F or S665V mutated version. Homologous recombination was achieved by recombinase-mediated cassette exchange as described before for other mutations of VE-cadherin (Fig. 1A) [8, 9]. Control experiments were performed with VE-cadherin-WT knock-in mice, where a wild-type VE-cadherin cDNA was targeted to the endogenous VE-cadherin locus. As previously shown, VE-cadherin expression levels and junctional localization are indistinguishable between wild-type C57BL/6 and VE-cadherin-WT knock in mice [18]. The VE-cadherin-Y658F and VE-cadherin-S665V point-mutated mice were viable, fertile, and healthy and developed normally. Heterozygous and homozygous mutant offspring were born according to the expected Mendelian ratio. Both mutated versions of VE-cadherin were expressed at the same level as VE-cadherin-WT, as determined by immunoblot analysis of whole lung lysates (Fig. 1B). Furthermore, both VE-cadherin point mutants displayed regular association with catenins, as similar levels of α-, β- and γ-catenin as well as p120-catenin coimmunoprecipitated with each of the VE-cadherin variants from lung lysates (Fig. 1C). Whole mount staining of the cremaster muscle with antibodies against VE-cadherin and PECAM-1 confirmed similar levels of expression and junctional recruitment for the two VE-cadherin variants as for WT VE-cadherin (Fig. 1D, E).

### Generation of antibodies specifically recognizing pY658 and pS665

**Fig. 2:**
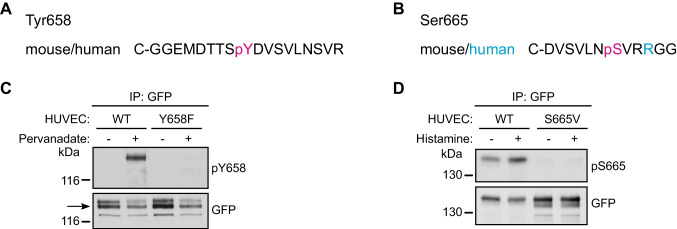
Generation and characterization of antibodies directed against pY658 and pS665. **A** Synthetic peptide corresponding to the sequence surrounding Tyr658 of VE-cadherin which was used to generate antibodies against phosphorylated Tyr658 (pY658). **B** Synthetic peptide corresponding to the sequence surrounding Ser665 of VE-cadherin which was used to generate antibodies against phosphorylated Ser665 (pS665) **C** Specificity of the anti-pY658 antibody in HUVEC. HUVEC were transduced with EGFP-tagged VE-cadherin WT or Y658F constructs. Cells were treated with pervanadate and immunoprecipitates of VE-cadherin-EGFP were immunoblotted for pY658 and GFP. **D** Specificity of the anti-pS665 antibody in HUVEC. HUVEC were depleted of endogenous VE-cadherin followed by re-expression of VE-cadherin-WT-EGFP or the corresponding S665V mutant. Cells were stimulated with histamine for 10 min and immunoprecipitates of VE-cadherin-EGFP were immunoblotted for pS665 and GFP. Molecular weight markers are indicated in kDa (C, D). Data are representative of at least *n = *3 independent experiments

In order to monitor the phosphorylation state of Y658 and S665 of VE-cadherin, we generated antibodies specific for the respective phosphorylated residues. The monoclonal antibody against pY658, L36.1, was produced by immunizing rats with a synthetic peptide corresponding to the sequence surrounding pY658 of human and mouse VE-cadherin (Fig. 2A), whereas the polyclonal anti-pS665 antibody, VD53, was produced in rabbits immunized with a phosphorylated peptide covering the sequence around S665 of human VE-cadherin (Fig. 2B). The specificity of both antibodies was tested in HUVEC transduced with adenoviral vectors expressing EGFP-tagged versions of WT or point-mutated VE-cadherin with either Y658 replaced by phenylalanine or S665 replaced by valine. Treatment of HUVEC with the tyrosine phosphatase inhibitor peroxyvanadate strongly increased the anti-pY658 immunoblot signal for VE-cadherin-WT-EGFP whereas no signal was seen for the VE-cadherin-Y658F-EGFP mutant (Fig. 2C). With the anti-pS665 antibody we found a weak baseline signal for VE-cadherin-WT-EGFP, which clearly increased in cells treated for 10 min with histamine, while no signal was obtained in HUVEC expressing VE-cadherin-S665V (Fig. 2D). These results demonstrate that the monoclonal anti-pY658 and the polyclonal anti-pS665 antibody are each specific for their respective phosphorylation site.

### VEGF- and histamine-induced vascular permeability in vivo requires S665 but not Y658 phosphorylation

**Fig. 3:**
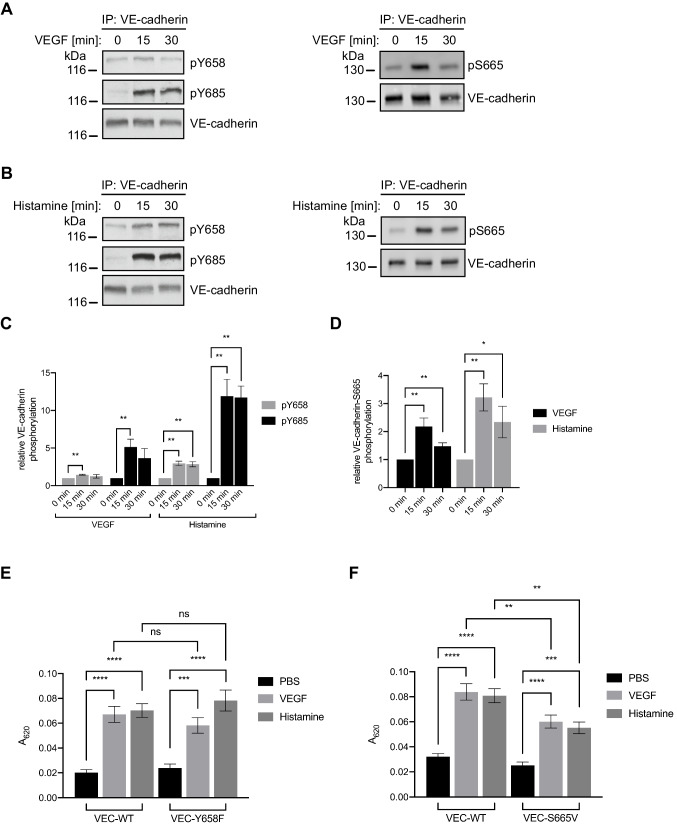
Permeability induction in the skin is not impaired in mice expressing VEC-Y658F, but in mice expressing VEC-S665V. **A** + **B** Immunoblot analysis of VE-cadherin immunoprecipitates from HUVEC stimulated for 15 or 30 min with VEGF (A) or histamine (B), detected with mAb pY658, mAb pY685, pAb pS665 and anti-VE-cadherin. **C**-**D** Quantification of immunoblot signals in (A) and (B), presented as relative VE-cadherin phosphorylation on (C) Y658 and Y685 and (D) S665, normalized to VE-cadherin-EGFP levels. **E** + **F** Miles Assay in skin of VEC-WT, VEC-Y658F or VEC-S665V mice given intravenous injection of Evans Blue dye and, 15 min later, intradermal injection of PBS, VEGF or histamine followed by quantification of dye extracted from excised skin areas 30 min later, shown as absorbance at 620 nm (A_620_). ****P < 0.0001; ***P < 0.001; **P < 0.01; ns, not significant (One-way ANOVA with Tukey’s multiple comparisons test). Data are representative of at least three independent experiments (A-D) or are from *n = *4 independent experiments with five mice per group and experiment (E, mean ± SEM of 20 mice) or from *n = *5 independent experiments with five-eight mice per group and experiment (F, mean ± SEM of 29–33 mice)

Previous studies on the role of phosphorylation of VE-cadherin on Y685 in vascular barrier regulation have shown that phosphorylation of this residue is upregulated by permeability-enhancing mediators such as VEGF and histamine in vitro [18]. It was furthermore demonstrated that stimulation with VEGF also induces phosphorylation of VE-cadherin on S665 [22]. To address whether this would hold true for the phosphorylation of Y658 as well, we compared the effect of VEGF and histamine stimulation of HUVEC on the phosphorylation state of Y685, Y658 and S665. As previously reported [18], baseline phosphorylation of Y685 was very low and strongly upregulated by both mediators after 15 and 30 min (Fig. 3A, B, C). Likewise, phosphorylation of S665 was strongly increased at 15 min, however declined by 30 min (Fig. 3A, B, D). In contrast to this, Y658 phosphorylation was only weakly increased by either of the stimuli at both investigated time points (Fig. 3A, B, C). Given the published necessity of Y685 phosphorylation for vascular permeability induction in vivo [18], we next investigated the functional relevance of Y658 and S665 phosphorylation for this process. To this end, we performed Miles Assays with S665V and Y658F mutant as well as VE-cadherin-WT knock-in mouse lines. Mice were intravenously injected with Evans Blue dye and given a local intradermal stimulus of PBS, VEGF or histamine 15 min later. Quantification of the extravasated dye after 30 min showed that the histamine and VEGF injection strongly induced vascular leaks in VE-cadherin-WT knock-in mice (Fig. 3E, F). Illustrative images are depicted in Supplementary Fig. 1. In mice expressing Y658F-mutated VE-cadherin, permeability was induced by both mediators with similar efficiency as in VE-cadherin-WT mice (Fig. 3E). In VE-cadherin-S665V mice, by contrast, the permeability increases induced by VEGF and histamine were significantly lower (30% and 26%, respectively) than for VE-cadherin-WT (Fig. 3F). Thus, phosphorylation of S665 but not Y658 contributes to the regulation of vascular permeability in vivo, which is in agreement with the observation that histamine and VEGF increase Y658 phosphorylation only very modestly, while S665 phosphorylation is strongly stimulated.

### Histamine-induced endocytosis of VE-cadherin requires S665 but not Y658 phosphorylation

**Fig. 4:**
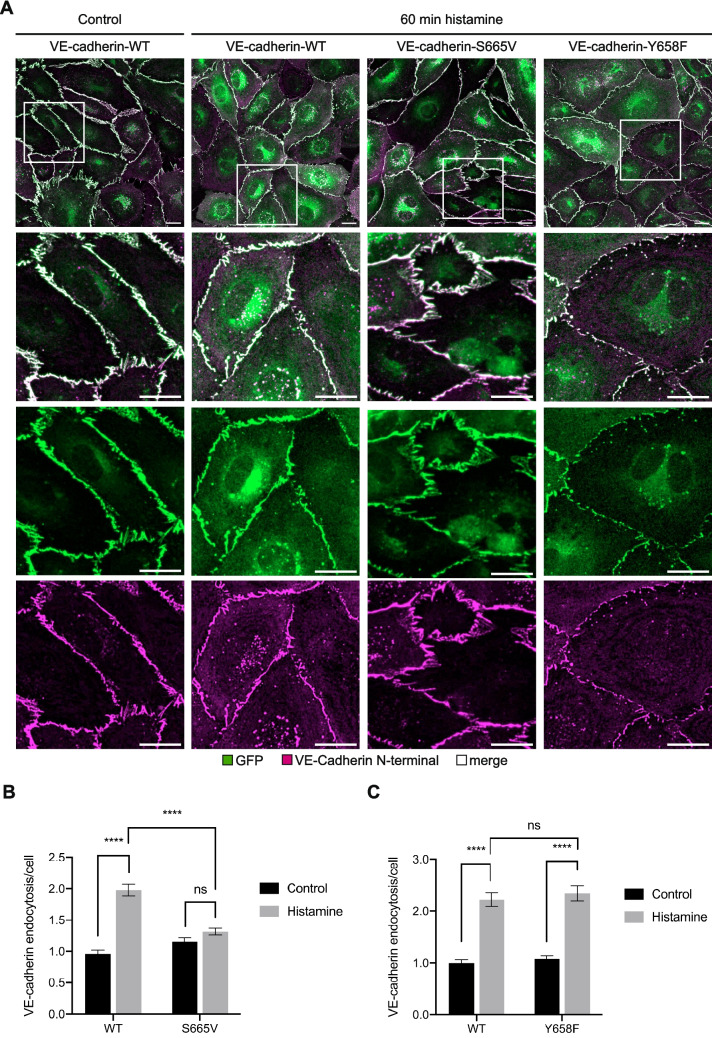
Phosphorylation at S665 but not Y658 is required for histamine-induced endocytosis of VE-cadherin. HUVEC were depleted of endogenous VE-cadherin by siRNA transfection followed 24 h later by adenoviral re-expression of VE-cadherin-WT-EGFP, -S665V-EGFP or -Y658F-EGFP. HUVEC were incubated with an Alexa Fluor 647-coupled anti-VE-cadherin antibody and 100 μM histamine or vehicle for 60 min and fixed for microscopic analysis. **A** Images were acquired with a Zeiss LSM 880 confocal microscope equipped with an Airyscan detector (20 × objective) and are shown as maximum intensity projections of Z-stacks. Scale bars are 20 μm. **B** + **C** The number of vesicles double positive for GFP and Alexa Flour 647 was counted manually, normalized to the number of cells evaluated and expressed relative to VE-cadherin-WT control. Data are from 20 images per condition per experiment, were pooled from *n = *3 independent experiments and are expressed as mean ± SEM. *** *P* < 0.0001; ns, not significant (Two-way ANOVA with Tukey’s multiple comparisons test)

It was shown previously that the Y685F point mutation of VE-cadherin is associated with a reduction of VEGF- and histamine-induced vascular permeability in vivo and that the VE-cadherin-Y685F mutant is resistant to internalization in HUVEC stimulated with histamine [18, 19]. To elucidate if the inhibitory effect of the VE-cadherin-S665V mutation on vascular permeability induction seen in Miles Assays might result from defective endocytosis of the VE-cadherin mutant, we tested the effect of the mutation on histamine-induced internalization of the protein. To this end, HUVEC were depleted of endogenous VE-cadherin by siRNA (Supplementary Fig. 2A) and transduced with WT VE-cadherin-EGFP or the corresponding S665V mutant. Cells were then incubated with histamine for 60 min together with a fluorophore-conjugated, non-adhesion blocking antibody against the VE-cadherin N-terminus (55-7H1). This enabled us to distinguish endocytosed VE-cadherin from newly synthesized, intracellular VE-cadherin on its way to the cell surface. Following fixation, endocytosis was quantified as the number of intracellular vesicles which were double positive for EGFP and staining with 55-7H1, and normalized to the number of cells evaluated. This revealed a significant increase in the number of endocytic vesicles upon histamine stimulation in cells expressing WT VE-cadherin, whereas in cells expressing VE-cadherin-S665V, internalization was not increased by histamine. Furthermore, the S665V mutation did not affect the steady-state level at which VE-cadherin was endocytosed, suggesting that constitutive turnover of VE-cadherin is independent of S665 phosphorylation (Fig. 4A, B). Since we found that the Y658F mutation did not protect VE-cadherin mutant knock-in mice against histamine- and VEGF-induced vascular permeability, we next analyzed whether this mutation would affect the internalization of VE-cadherin (VE-cadherin silencing shown in Supplementary Fig. 2B). In line with the in vivo data, endocytosis of VE-cadherin was increased upon histamine stimulation to the same extent in cells expressing VE-cadherin-Y658F as in cells expressing the WT construct (Fig. 4A, C). Together, these results suggest that impaired induction of vascular permeability in VE-cadherin-S665V mutant knock-in mice results from an inhibitory effect of the mutation on VE-cadherin endocytosis induced by inflammatory mediators, whereas phosphorylation of Y658 is not relevant for this process

### S665 phosphorylation precedes that of Y685

**Fig. 5:**
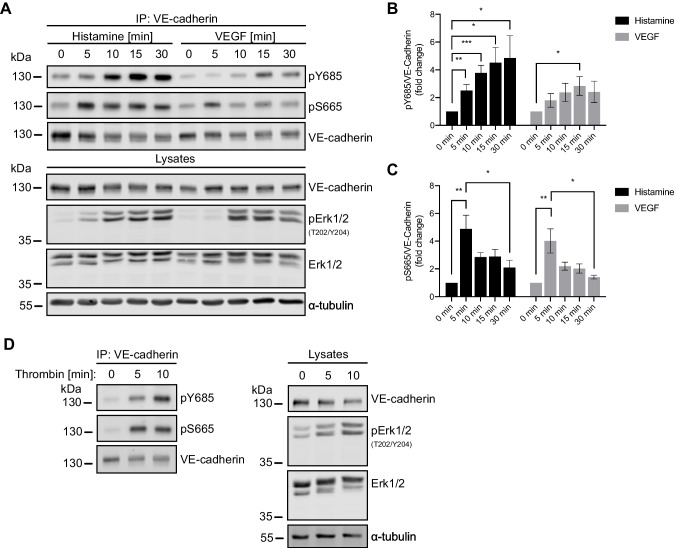
Phosphorylation of S665 and Y685 of VE-cadherin follow distinct kinetics. **A** HUVEC were serum-starved overnight followed by stimulation with histamine or VEGF for 5, 10, 15 or 30 min. Cells were lysed and VE-cadherin immunoprecipitates were blotted for pY685, pS665 and VE-cadherin. Cell lysates were analyzed by Western Blot with antibodies against VE-cadherin, pErk1/2 and Erk1/2, using α-tubulin as loading control. Molecular weight markers are indicated in kDa. Phosphorylation of **B** Y685 and **C** S665 was normalized to VE-cadherin input, quantified from *n = *6 independent experiments and expressed as mean ± SEM. *** *P* < 0.001, ** *P* < 0.01, * *P* < 0.05 (unpaired two-tailed t-test). **D** HUVEC were serum-starved for 3 h followed by stimulation with thrombin for 5 min or 10 min. Cells were lysed and VE-cadherin immunoprecipitates were blotted for pY685, S665 and VE-cadherin. Cell lysates were analyzed by Western Blot with antibodies against VE-cadherin, pErk1/2 and Erk1/2, using α-tubulin as loading control. Molecular weight markers are indicated in kDa (A, D)

When comparing the phosphorylation of S665 and Y685 in HUVEC stimulated with histamine or VEGF, we found that the phosphorylation of VE-cadherin on Y685 was sustained over the 30 min observation period, whereas that of S665 was increased at 15 min but declined again at 30 min (Fig. 3A, B). This prompted us to directly compare the phosphorylation kinetics of each site in more detail. To this end, HUVEC were treated with histamine or VEGF and lysed after 5, 10, 15 or 30 min (Fig. 5A). Stimulation efficacy was verified by probing total cell lysates for phosphorylated T202 and Y204 of extracellular signal-regulated kinase 1 and 2 (pErk1/2), which are known to be activated both downstream of histamine and VEGF [27, 28]. Immunoblot analyses of VE-cadherin immunoprecipitates revealed that for both stimuli, Y685 phosphorylation increased gradually, was maximal after 15 min, and sustained over 30 min (Fig. 5B). By contrast, the phosphorylation level of S665 reached its maximum after 5 min and began to drop at 10 min (Fig. 5C). This suggested that phosphorylation of VE-cadherin on these two residues follows distinct kinetics, with phosphorylation of S665 preceding that of Y685, but that of Y685 being sustained for a longer time period.

Because phosphorylation of Y685 and S665 are both triggered by VEGF and histamine, we sought to determine whether thrombin, another potent mediator of vascular permeability, also induces phosphorylation of these residues. We therefore stimulated HUVEC with thrombin for 5 or 10 min followed by immunoprecipitation of VE-cadherin from cell lysates. Immunoblot analysis confirmed that both phosphorylation sites are also targeted downstream of thrombin (Fig. 5D). Thus, phosphorylation of S665 and Y685 is a common phenomenon to several inflammatory mediators, among these VEGF, histamine and thrombin, all of which are known to induce vascular permeability [29–31].

### Y685 and S665 phosphorylation are independent events

**Fig. 6:**
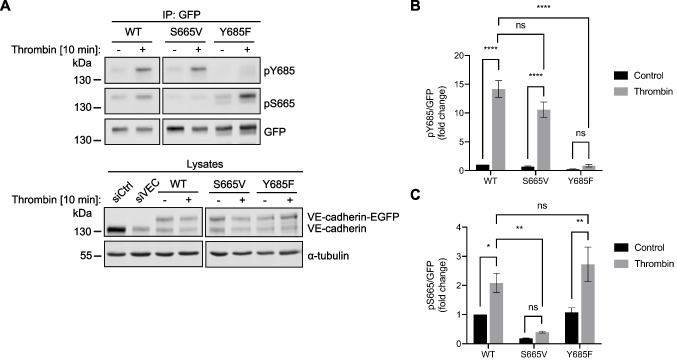
Phosphorylation of S665 and Y685 of VE-cadherin are independent of one another. **A** HUVEC were transfected with control (siCtrl) or VE-cadherin (siVEC) siRNA followed 24 h later by adenoviral reexpression of VE-cadherin-WT-EGFP, -S665V-EGFP and -Y685F-EGFP. Cells were serum-starved for 3 h, treated with Na_3_VO_4_ and NaF for 20 min and stimulated with thrombin for 10 min. VE-cadherin was precipitated from cell lysates with an anti-GFP antibody. Precipitates were immunoblotted using antibodies against pY685, pS665 and GFP. Cell lysates were blotted for VE-cadherin, using α-tubulin as loading control. Molecular weight markers are indicated in kDa. Phosphorylation of **B** Y685 and **C** S665 was normalized to VE-cadherin-EGFP input, quantified from *n = *4 independent experiments and expressed as mean ± SEM. **** *P* < 0.0001, ** *P* < 0.01, * *P* < 0.05; ns, not significant (Two-way ANOVA with Tukey’s multiple comparisons test)

Since we found S665 phosphorylation to be induced more rapidly but less long-lasting than that of Y685, we next analyzed whether phosphorylation of both sites might be a sequential process, such that phosphorylation of one site would be a prerequisite for phosphorylation of the other. To this end, we silenced endogenous VE-cadherin in HUVEC by siRNA transfection followed by adenoviral expression of VE-cadherin-WT-EGFP or the corresponding EGFP-tagged VE-cadherin-Y685F or -S665V mutants, respectively. Immunoblot analysis of GFP immunoprecipitates indicated that stimulation of cells with thrombin led to an increase in VE-cadherin phosphorylation on Y685 that was not affected by the mutation of S665 (Fig. 6A, B). Likewise, S665 phosphorylation was induced to the same extent in cells expressing VE-cadherin-WT or VE-cadherin-Y685F (Fig. 6A, C). We conclude that despite the distinct kinetic patterns, phosphorylation of Y685 and S665 are independent processes.

## Discussion

In this study, we investigated whether the phosphorylation of VE-cadherin at S665 and Y658 is in vivo relevant for the regulation of vascular permeability in the context of inflammation. Generating point-mutated VE-cadherin knock-in mice, we found that phosphorylation of S665 was required for the induction of vascular permeability by histamine and VEGF in the skin, whereas phosphorylation of Y658 was dispensable for this process. Likewise, only the S665V mutation blocked histamine-induced internalization of VE-cadherin in HUVEC, whereas no such effect was seen for the Y658F mutation. Comparing the kinetics of S665 phosphorylation with that of Y685, another important residue implicated in the regulation of vascular leak formation [18], we found S665 to be phosphorylated more rapidly, while phosphorylation of Y685 was sustained longer. Nevertheless, each of these two residues was phosphorylated independent of the phosphorylation state of the other. Collectively, our data suggest that phosphorylation of Y658 is irrelevant for inflammation-induced endothelial permeability in the context of several tested mediators, and substantiate S665 as an important site regulating VE-cadherin internalization in vitro and vascular leak formation in vivo independently of Y685.

Besides several cytoplasmic tyrosine residues of VE-cadherin, S665 was identified as a major phosphorylation site promoting VEGF-induced VE-cadherin endocytosis and endothelial permeability in vitro [22]*.* Mechanistically, S665 phosphorylation was shown to be mediated by a complex signaling cascade comprising Src kinase, the Rho-GEF Vav2, the small GTPase Rac1 and the kinase PAK1, leading to the recruitment of the cytosolic scaffolding protein β-arrestin2 and thereby the internalization of VE-cadherin [22]. However, the relevance of S665 phosphorylation for the control of the endothelial barrier was never studied in vivo, and little is known about the dynamic interplay of this site with regulatory tyrosine residues of VE-cadherin and their individual contribution to vascular permeability induction. In agreement with the report that VEGF induces VE-cadherin endocytosis via phosphorylation of S665, we found that phosphorylation of S665 was also triggered by thrombin and histamine, and was required for the endocytosis of VE-cadherin in histamine-stimulated endothelial cells. Furthermore, the VE-cadherin-S665V mutation in our knock-in mouse line reduced VEGF- and histamine-induced plasma leaks in the skin. Thus, S665 of VE-cadherin is a crucial phosphorylation site regulating inflammation-induced vascular permeability in vivo*.*

It is intriguing that the relevance of S665 for the induction of VE-cadherin endocytosis by VEGF [22] and histamine (this study) in cultured human endothelial cells in vitro could be fully matched by the relevance of this site for vascular permeability induction in the mouse in vivo. The same concordance between in vitro findings in human endothelial cells and in vivo observations in the mouse was found for Y685 and Y731, with the first being important for vascular permeability induction, and the latter for leukocyte transmigration [18, 19, 32]. Collectively, this agreement between in vitro and in vivo findings across species highlights the physiological relevance of the modification of these sites in VE-cadherin for the control of endothelial junctions.

Interestingly, the S665V mutation completely prevented VE-cadherin endocytosis induced by histamine, whereas the inhibitory effect on vascular permeability was not complete. As we have shown previously, the same is true for the Y685F mutation, which completely abolished VE-cadherin endocytosis but only partially reduced vascular leaks in the skin [18, 19]. Thus, phosphorylation of S665 and Y685 are each necessary but not sufficient for the induction of vascular permeability by inflammatory mediators. This implies that additional mechanisms beyond VE-cadherin endocytosis must exist which contribute to the opening of endothelial junctions and the formation of vascular leaks. It is intriguing to speculate that such additional mechanisms may somehow regulate the adhesive activity (*trans* interactions) of VE-cadherin molecules. *Trans* interactions of VE-cadherin molecules could be affected by mechanisms addressing the cadherin–catenin–actin linkage [10–12] or by interference with the conformation of the extracellular part of VE-cadherin. Changes of the conformation of E-cadherin induced by antibodies were indeed shown to modulate the adhesive activity of this cadherin [33] and similar effects were shown for VE-cadherin [34]. Potential mechanisms that might directly weaken the adhesive function (*trans* binding affinity/avidity) of VE-cadherin would probably increase the number of VE-cadherin molecules available for endocytosis. In combination with effects that increase the endocytosis process, such mechanisms might synergize in the process of junction destabilization.

Previously, we have shown that histamine and VEGF induce phosphorylation of Y685 of VE-cadherin in vitro and in vivo, and that VE-cadherin-Y685F mutant knock-in mice exhibit reduced vascular permeability induction in the skin in response to both of these inflammatory stimuli [18]. Likewise, the VE-cadherin-Y685F mutant was protected from histamine-induced endocytosis in vitro [19]. Thus, the Y685F and S665V mutations had strikingly similar functional consequences, and both of them completely prevented inflammation-induced VE-cadherin internalization. This prompted us to investigate whether phosphorylation of these two sites might contribute to VE-cadherin endocytosis via the same pathway. Importantly, we found the kinetics of S665 and Y685 phosphorylation to be distinctly different, with maximal phosphorylation of S665 preceding that of Y685, while Y685 phosphorylation remained at a high level for a longer time period. This opened the possibility that phosphorylation of both residues might occur sequentially. Surprisingly, however, we found that in HUVEC expressing VE-cadherin-S665V, phosphorylation of Y685 was still inducible by thrombin, and the same held true for phosphorylation of S665 in VE-cadherin-Y685F. The fact that S665 and Y685 phosphorylation follow distinct kinetics may suggest that both residues cooperate to fine-tune the kinetics of VE-cadherin internalization. Alternatively, the need for two distinct phosphorylation events to trigger VE-cadherin endocytosis and thereby induce vascular permeability might serve as a safeguard mechanism that prevents premature opening of junctions, such that phosphorylation of one site alone would not be sufficient to affect the integrity of the endothelial barrier.

The remarkable finding that phosphorylation of S665 and Y685 are processes that regulate vascular permeability independently but in parallel is supported by previous studies which showed that both events culminate in the same pathway leading to VE-cadherin endocytosis. pS665 was reported to recruit the scaffolding protein β-arrestin2 [22], and inflammation-induced phosphorylation of Y685 has been linked to VE-cadherin ubiquitination on lysine residues 626 and 663 [17, 19]. Each of these events, β-arrestin-2 binding and ubiquitination, are known to trigger internalization of proteins via clathrin-mediated endocytosis.

The fact that three different residues of VE-cadherin are target for junction-regulating mechanisms and each is subject for another internalization-regulating pathway [19, 22, 32] raises the question why VE-cadherin is such a central aim for a multitude of endocytosis mechanisms. Could it be that the different phosphorylation sites on VE-cadherin serve as functional interfaces linking it to other junctional proteins, thereby orchestrating the formation of distinct signaling complexes in response to specific stimuli? We have found previously that ESAM supports neutrophil extravasation and VEGF-induced vascular permeability in vivo [23]. Preliminary, recently obtained unpublished results suggest that ESAM supports VEGF-induced phosphorylation of Y685. It will be interesting to investigate in the future whether ESAM indeed supports inflammation-induced junction destabilization by contributing to the modification of VE-cadherin. In addition, further members of junctional adhesion receptors should be analyzed in this context.

While the importance of Y685 for the control of vascular permeability is well documented [16–18, 35, 36], the relevance of Y658 as a regulatory phosphorylation site is controversial. Stimulation of VE-cadherin endocytosis by histamine and bradykinin were inhibited by the Y658F point mutation and also bradykinin-induced paracellular permeability in vitro was significantly reduced by this mutation [17]. Similarly, VEGF-induced permeability in vitro was inhibited by VE-cadherin-Y658F [37], and TNF-α-induced loss of electrical resistance across endothelial cell monolayers was blocked by this mutant [38]. In contrast, another study found no inhibitory effect of VE-cadherin-Y658F in human endothelial cells on VEGF-induced permeability in vitro [20]. This ambiguity of published results and the limitations of mimicking fully mature endothelial junctions in cultured endothelial cells made it necessary to investigate the role of VE-cadherin-Y658 in vivo. Our finding that the VE-cadherin-Y658F mice were not protected from VEGF- or histamine-induced hyperpermeability in the skin was in line with our in vitro results showing that neither of these stimuli increased Y658 phosphorylation considerably, nor was histamine-induced VE-cadherin endocytosis inhibited by the Y658F mutation. Together, our results suggest that phosphorylation of Y658 does not contribute to vascular permeability induction under inflammatory conditions in vivo.

Instead, Y658 of VE-cadherin is relevant for the response of endothelial cells to fluid shear stress. It was shown that VE-cadherin is constitutively phosphorylated at Y658 in venules and capillaries but not in arteries [17]. The same study showed that low fluid shear stress induced the phosphorylation of VE-cadherin-Y658. A more detailed analysis revealed that low or oscillatory flow induced phosphorylation of Y658 in a small pool of VE-cadherin molecules, which caused dissociation of p120-catenin which then was replaced by the polarity protein LGN [39]. This study also showed that VE-cadherin-Y658F mice, which we describe and characterize here for the first time, were impaired in flow-dependent vascular remodeling in a femoral artery ligation model [39]. Mechanistically, it was suggested that downstream of flow-induced Y658 phosphorylation and of LGN, proinflammatory signaling via NF-κB was activated [39], a well-described target of flow signaling by the PECAM-1/VE-cadherin/VEGFR junctional mechanosensory complex [40]. Thus, phosphorylation of VE-cadherin-Y658 is an important step in flow-induced pro-inflammatory signaling, yet not in inflammatory responses that trigger vascular permeability by mediators such as histamine or VEGF, as we show here.

Collectively, our study rules out the involvement of VE-cadherin-Y658 phosphorylation for vascular leak formation and establishes S665 of VE-cadherin as an important phosphorylation site regulating inflammation-induced vascular permeability in vivo. We provide evidence that phosphorylation of S665 and Y685 of VE-cadherin, which are initiated downstream of several inflammatory mediators, are independent yet parallel events that together contribute to the internalization of VE-cadherin from endothelial cell junctions.

## Supplementary Information

Below is the link to the electronic supplementary material.

Supplementary file 1 (PDF 232 KB) Visual representation of Miles Assay skin areas. Miles Assays were performed with VEC-WT, VEC-Y658F or VEC-S665V mice as described in Fig. 3 E, F. Images are representative visualizations of the inside of the excised back skin of one animal per group. PBS: PBS control injection site, VEGF: VEGF injection site, His: histamine injection site.

Supplementary file 2 (PDF 1373 KB) Endogenous VE-cadherin silencing efficiencies. HUVEC were transfected with control (siCtrl) or VE-cadherin (siVEC) siRNA. Cell lysates were immunoblotted for endogenous VE-cadherin and α-tubulin. Molecular weight markers are indicated in kDa. Silencing efficiencies in (A) and (B) refer to the experiments shown in Fig. 4 A, B and Fig. 4 A, C, respectively.

## Data Availability

This study includes no data deposited in external repositories. All data supporting the findings of this study are available within the paper and its Supplementary Information.
