# Supplementary figures and images for: Distinct VE-cadherin serine and tyrosine phosphorylation sites and their role for inflammation-induced vascular permeability in vivo
Source: Cell Mol Life Sci. 2025 Jun 5;82(1):223. doi: 10.1007/s00018-025-05753-2 (PMC12141182; doi:10.1007/s00018-025-05753-2)

# Supplementary Figure 1

VE-cadherin-WT

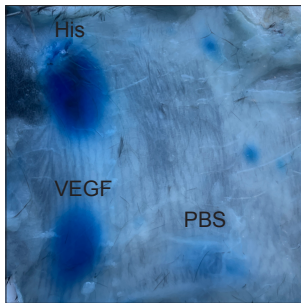

VE-cadherin-Y658F

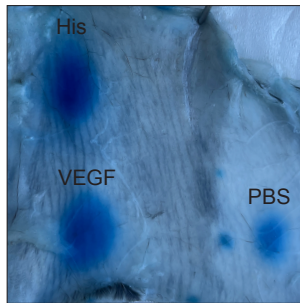

VE-cadherin-S665V

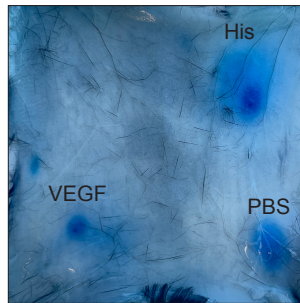

Supplement: Supplementary file 1 — (PDF 232 KB) Visual representation of Miles Assay skin areas. Miles Assays were performed with VEC-WT, VEC-Y658F or VEC-S665V mice as described in Fig. 3 E, F. Images are representative visualizations of the inside of the excised back skin of one animal per group. PBS: PBS control injection site, VEGF: VEGF injection site, His: histamine injection site. [file 18_2025_5753_MOESM1_ESM.pdf]

# Supplementary Figure 2

**A**

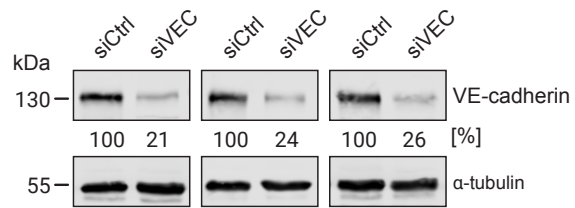

**B**

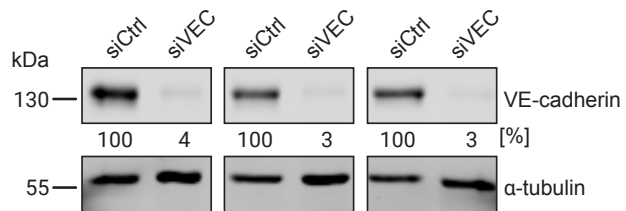

Supplement: Supplementary file 2 — (PDF 1373 KB) Endogenous VE-cadherin silencing efficiencies. HUVEC were transfected with control (siCtrl) or VE-cadherin (siVEC) siRNA. Cell lysates were immunoblotted for endogenous VE-cadherin and α-tubulin. Molecular weight markers are indicated in kDa. Silencing efficiencies in (A) and (B) refer to the experiments shown in Fig. 4 A, B and Fig. 4 A, C, respectively. [file 18_2025_5753_MOESM2_ESM.pdf]
